# Paclitaxel- and Sirolimus-Coated Balloons Versus Drug-Eluting Stents in Coronary Artery Disease: A Comprehensive Narrative Review

**DOI:** 10.3390/life16010063

**Published:** 2025-12-31

**Authors:** Flavius-Alexandru Gherasie, Al Hassan Ali, Ana Maria Corzanu, Eva Catalina Costescu, Sonia-Gabriela Cornea

**Affiliations:** 1 Department of Cardiology, University of Medicine and Pharmacy “Carol Davila,” 050474 Bucharest, Romania; anacorzanu@gmail.com (A.M.C.); eva.costescu@yahoo.com (E.C.C.); soniagabrielacornea@gmail.com (S.-G.C.); 2 Emergency Clinical Hospital Dr. Bagdasar-Arseni, 050474 Bucharest, Romania; 3 Department of Cardiology, Elias University Emergency Hospital, 011461 Bucharest, Romania

**Keywords:** drug-coated balloon, drug-eluting stent, in-stent restenosis, paclitaxel, sirolimus, coronary artery disease, percutaneous coronary intervention, target lesion revascularization, major adverse cardiac events, acute coronary syndrome, late lumen loss, minimal lumen diameter, balloon angioplasty

## Abstract

Drug-coated balloons (DCBs) have emerged as an alternative to drug-eluting stents (DESs) in percutaneous coronary intervention, delivering antiproliferative drugs without leaving a permanent implant. This review provides a comparative analysis of sirolimus-coated DCBs (DCB-S), paclitaxel-coated DCBs (DCB-P), and DESs across key scenarios: de novo coronary lesions in chronic coronary syndrome (CCS), acute coronary syndromes (ACS), and in-stent restenosis (ISR). We discuss late lumen loss (LLL), target lesion/vessel revascularization (TLR/TVR), vessel patency, and major adverse cardiac events (MACE) outcomes, along with current guidelines and emerging indications for DCB-S. We also examine pharmacological differences between sirolimus and paclitaxel (mechanisms of action, tissue uptake, and healing profiles), trial methodologies, and recent innovations in DCB technology. Across stable de novo lesions (especially small vessels and high bleeding-risk patients), multiple trials show DCB-P can achieve non-inferior clinical outcomes to DES. Early data suggest newer DCB-S may likewise match DES outcomes in broader populations. In ACS, DCB-only strategies have demonstrated feasibility and safety in carefully selected lesions without heavy thrombus, with randomized studies like REVELATION (STEMI) showing non-inferior fractional flow reserve and low revascularization rates compared to DES. For ISR, DCB-P is an established Class I treatment in both BMS-ISR and DES-ISR, yielding similar or lower TLR rates than repeat stenting. DCB-S are now being evaluated as an alternative in ISR, aiming to avoid additional stent layers. Contemporary guidelines endorse DCB use in ISR and small vessels, and experts anticipate expanding indications as evidence grows. Sirolimus and paclitaxel differ in antiproliferative mechanisms and pharmacokinetics—sirolimus (cytostatic, mTOR inhibition) may offer faster endothelial recovery, whereas paclitaxel’s high lipophilicity ensures sustained arterial wall retention. Technological advances (e.g., phospholipid micro-reservoirs for sirolimus) are enhancing drug transfer and addressing prior limitations. In summary, DCB-P and DCB-S now represent viable alternatives to DES in specific scenarios, especially where “leaving nothing behind” could reduce long-term complications. Ongoing large randomized trials, such as SELUTION DeNovo, currently available as conference-presented data, together with longer-term follow-up will further clarify the optimal niches for DCB-S versus DCB-P and DES.

## 1. Introduction

Drug-eluting stents (DESs) and drug-coated balloons (DCBs) are percutaneous coronary devices designed to prevent restenosis following angioplasty. DESs consist of a metallic scaffold coated with a polymer that releases an antiproliferative drug over time, whereas DCBs deliver the drug directly to the vessel wall during balloon inflation without leaving a permanent implant [1,2,3,4]. Key device characteristics such as material composition, size, structural design, and durability influence vascular healing and long-term outcomes [5,6].

Drug-eluting stents have revolutionized coronary revascularization by reducing restenosis, but their metallic scaffolds carry risks of chronic inflammation, neoatherosclerosis, and stent thrombosis [7,8,9,10]. To mitigate these issues, drug-coated balloons were developed as a “stent-less” approach: during angioplasty, an antiproliferative drug is delivered to inhibit neointimal hyperplasia, yet no permanent implant remains. By avoiding metal/polymer, DCBs may reduce late foreign-body reactions and allow arteries to heal more naturally [11]. Initial DCB applications focused on ISR, where adding another stent was undesirable. Pivotal trials (e.g., PACCOCATH, PEPCAD) confirmed that paclitaxel-coated balloons significantly reduce LLL and revascularization compared to plain balloon angioplasty in ISR [12,13]. Subsequently, DCBs have been explored in de novo lesions, particularly for small vessels and patients at high bleeding risk, with encouraging outcomes [2].

Paclitaxel has been the dominant DCB drug due to its lipophilicity and prolonged tissue retention, enabling effective one-time delivery. Recently, sirolimus (and analogues)—the workhorse drug on modern DES—is being applied to balloons. Sirolimus-coated DCBs offer a cytostatic mechanism that is potentially more favorable for endothelial healing but require innovative delivery systems (nanocarriers, micro-depots) to compensate for lower tissue uptake [14]. The SELUTION SLR (sirolimus) balloon, for example, uses phospholipid-coated micro-reservoirs for sustained 90-day drug release [3]. Early trials suggest that with these technologies, sirolimus DCBs can achieve therapeutic tissue levels and clinical results comparable to paclitaxel DCBs [5].

This article reviews DCB-S, DCB-P, and DES in three contexts: (1) de novo lesions in CCS and efficacy in stable CAD, (2) ACS as it pertains to treating culprit lesions in unstable settings, and (3) ISR as it relates to managing restenosis within stents. We synthesize evidence from trials and meta-analyses for late lumen loss, TLR, patency, and MACE, and we outline current guideline positions. We also provide tables summarizing key studies and visual comparisons including forest plots and schematic mechanism diagrams. An expanded discussion of trial methodologies, clinical relevance, and limitations is included, as well as emerging data on DCB-S platforms. The aim is to clarify where DCB-S and DCB-P stand today relative to DES and how pharmacologic differences translate into clinical strategy in interventional cardiology.

## 2. Pharmacologic Mechanisms of Paclitaxel vs. Sirolimus in DCBs

**Figure 1:**
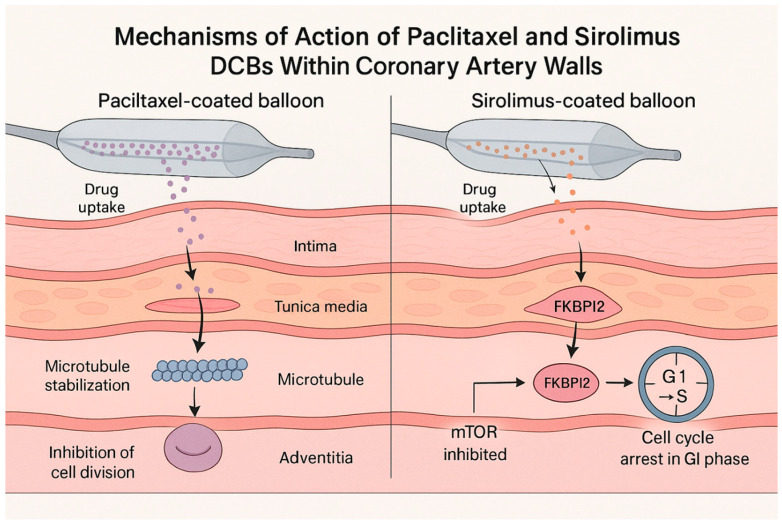
Mechanisms of action of paclitaxel vs. sirolimus from drug-coated balloons in the arterial wall.

Paclitaxel and sirolimus halt neointimal growth via distinct pathways. Paclitaxel (a cytotoxic diterpenoid) stabilizes microtubules and arrests cell cycling in G_2_/M transition, promoting apoptosis of vascular smooth muscle cells (Figure 1) [15]. Its high lipophilicity (logP~3.96) enables rapid uptake into the vessel wall and prolonged retention; the drug remains in tissue for weeks after a single inflation. This property underpins paclitaxel DCB efficacy despite brief balloon contact. However, paclitaxel’s cytotoxicity can also delay endothelial regrowth, contributing to moderate inflammation in some cases [15]. By contrast, sirolimus (rapamycin) is cytostatic, binding FKBP12 to inhibit the mTOR pathway and causing cell cycle arrest in G_1_ [16]. Sirolimus is less lipophilic (log *p*~2.5) and has a shorter tissue half-life than paclitaxel. Thus, early sirolimus DCB prototypes struggled to achieve adequate arterial drug levels. New excipient matrices (e.g., butyryl-hexyl citrate, phospholipids, or polymer microcapsules) have been developed to enhance sirolimus transfer and sustain release. Preclinical studies suggest that sirolimus-coated balloons may allow faster endothelial healing and attenuate inflammatory responses compared to paclitaxel [16]. Indeed, endothelial coverage was more complete and neointimal inflammation was milder with sirolimus in some models [16].

That said, clinical data so far do not show a definitive advantage of sirolimus DCBs in reducing restenosis or adverse events over paclitaxel DCBs; both drug coatings have shown broadly similar efficacy in trials [17]. The SIRPAC study, for example, found no significant difference in 12-month outcomes between a sirolimus DCB and a paclitaxel DCB [18]. Ongoing comparisons will further elucidate if the theoretical safety edge of sirolimus translates into improved clinical results. Table 1 outlines key pharmacologic differences between paclitaxel and sirolimus in DCB use.

Paclitaxel’s efficacy as a local antiproliferative was first shown in the 1990s when it inhibited smooth muscle proliferation in vitro and in injured arteries. This led to the development of paclitaxel-eluting stents and then paclitaxel DCBs. In 2004, Scheller et al. introduced the prototype paclitaxel-coated balloon, demonstrating marked neointimal suppression in porcine coronaries [19]. Clinically, the first randomized trial of DCB (PACCOCATH) in ISR showed a dramatic reduction in late lumen loss (0.03 mm with DCB vs. 0.74 mm with plain balloon), translating to fewer repeat revascularizations [12]. Sirolimus, conversely, was the drug on first-generation DESs (Cypher); its local vascular effects were well-characterized by 2001. It nearly eliminates restenosis when continuously eluted from stents. The challenge for DCB-S was achieving enough tissue drug retention from a short inflation. Through refined excipients, contemporary DCB-S now show tissue levels and vascular responses approaching those of limus-eluting stents [20]. For example, the MagicTouch DCB uses nanocarriers to deliver sirolimus effectively in human coronary lesions, yielding mid-term lumen enlargement rather than loss [20]. Thus, while paclitaxel remains the staple for DCBs (due to its proven performance and simplicity of formulation), sirolimus-based balloons are rapidly gaining traction as technology and evidence evolve.

## 3. DCB vs. DES in De Novo Coronary Lesions (Chronic Coronary Syndrome)

Background: DES implantation is standard for de novo lesions, but DCB-only intervention is an attractive option in certain scenarios (small vessels where stents perform suboptimally, diffuse disease requiring long stents, patients needing to avoid prolonged DAPT (e.g., high bleeding risk), and lesions in which preserving future treatment options is desirable). Without a stent, issues like chronic metal reaction, very late thrombosis, or artifact on imaging are eliminated [21]. Early attempts to use DCBs in de novo lesions had mixed results. The initial PICCOLETO trial (2009, paclitaxel DCB vs. BMS in small vessels) showed higher late loss and MACE with first-generation DCB, likely due to suboptimal balloon design and technique [22]. This tempered enthusiasm. However, subsequent trials with improved DCB technology and rigorous lesion preparation have been more positive [1,2].

**Figure 2:**
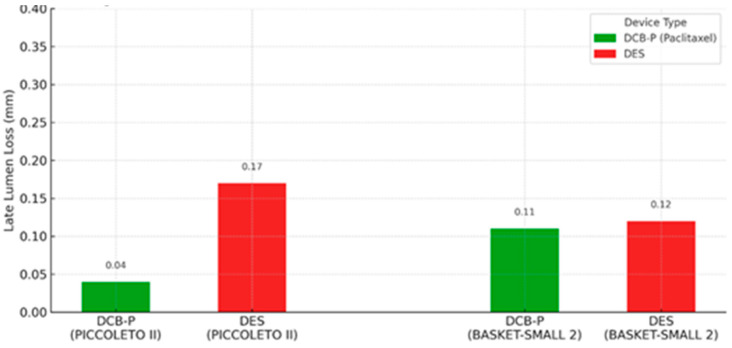
Late lumen loss at 6 months follow-up in PICCOLETO II and BASKET-SMALL 2 trials, comparing DCB-P vs. DES [23,24].

In terms of clinical trials, the landmark BASKET-SMALL 2 trial (2018) was a multicenter non-inferiority RCT in 758 patients with small vessels (<3 mm). After successful pre-dilation, patients were randomized to DCB (paclitaxel-coated) or second-generation DESs. At 12 months, DCB was non-inferior to DES for MACE (7.5% vs. 7.3%), with nearly identical Kaplan–Meier curves [1]. Three-year follow-up confirmed no difference in MACE (15% in both groups) and no excess thrombosis with DCB [23]. Notably, about half of DCB patients avoided any stent (“pure” DCB strategy), and outcomes were similar even in those with acute coronary syndrome presentations. As demonstrated in the BASKET-SMALL 2 trial, paclitaxel-coated balloons achieved non-inferior outcomes compared with DESs in small-vessel disease. Similarly, the PICCOLETO II trial (2020) focused on de novo small vessels (≤2.5 mm) and compared a new-generation paclitaxel DCB vs. an everolimus DES. At 6 months, the primary endpoint of in-lesion late lumen loss strongly favored DCB (0.04 mm vs. 0.17 mm with DES; *p* = 0.03), meeting criteria for both non-inferiority and statistical superiority. The DCB group also had lower binary restenosis (5% vs. 14%) and numerically fewer TLRs [2]. By 3 years, the trial showed a significant reduction in MACE with DCB (10.0% vs. 22.0% with DES) [24]. These results suggest that, in appropriately selected small-vessel lesions, paclitaxel DCB can match or even outperform DES in maintaining vessel patency. Figure 2 illustrates the difference in late lumen loss between DCB and DES observed in PICCOLETO II and BASKET-SMALL 2 trials.

Other studies reinforce these findings. The single-center RESTORE SVD trial (2019) reported that a paclitaxel DCB was non-inferior to DES in 1-year minimal lumen diameter for lesions ≤2.75 mm, with no difference in clinical outcomes [25]. Moreover, using DCB avoids multiple stents in long lesions—positive remodeling over time can even lead to late lumen enlargement (a phenomenon of vessel recoil reversal and plaque passivation). Paclitaxel’s effect of inducing extracellular matrix turnover is one hypothesized mechanism for late lumen gain in some DCB-treated segments. Yamamoto et al. observed this in 2020: DCB-treated de novo lesions show late lumen increase at 9 months (mean +0.13 mm), whereas DES segments typically show late loss [26].

Regarding sirolimus DCB in de novo lesions, until recently, virtually all clinical data in de novo coronaries were with paclitaxel DCBs. New trials are now evaluating sirolimus-coated balloons. One important study is a multicenter RCT by Ninomiya et al. (published 2023), which directly compared a sirolimus DCB to a paclitaxel DCB in de novo lesions. This trial (117 lesions, ~70% small vessels) found that at 6 months, angiographic outcomes favored the paclitaxel balloon: minimal lumen diameter was larger with DCB-P, and SCB failed to meet non-inferiority for in-segment late loss [27]. Restenosis occurred more than twice as often with the sirolimus DCB (33% vs. 12% with paclitaxel). While limited by the small sample, this suggests that first-generation SCBs have lower efficacy, likely due to inadequate drug transfer. However, not all SCBs are equal; newer devices with enhanced delivery (e.g., Selution SLR, MagicTouch) are showing better performance. Preliminary results from the SELUTION DeNovo trial, presented at major international meetings, evaluated a sirolimus-coated balloon strategy with provisional stenting versus routine DES implantation in de novo coronary lesions. The trial met criteria for non-inferiority at 1 year; however, these findings should be interpreted with caution until a full peer-reviewed publication becomes available. Accordingly, these data are considered hypothesis-generating and supportive rather than definitive [3]. In 3323 patients (vessel diameters 2–5 mm, excluding left main or CTOs), the 1-year target-vessel failure was 5.3% for the DCB strategy vs. 4.4% for DES.

There were no significant differences in cardiac death or MI, and clinically-driven TLR was low in both arms (3.3% DCB vs. 2.1% DES). Approximately 20% of patients in the DCB arm received a bailout stent, usually for significant recoil or dissection. Interestingly, subgroup analyses hinted that DCB might be especially beneficial in certain groups (e.g., patients with high bleeding risk or heavily calcified lesions had numerically lower TVF with DCB). Although DCB underperformed in women in that study for unclear reasons, the overall takeaway is that a sirolimus DCB strategy can be as safe and effective as routine DES implantation in many de novo lesions [5]. This represents a paradigm shift, as it broadens DCB use beyond just small vessels. Nevertheless, long-term follow-up (beyond 1 year) will be crucial to confirm durability and late event rates.

Regarding clinical considerations, the successful use of DCB in de novo lesions hinges on meticulous technique. Lesion preparation is critical; a high-pressure pre-dilation or atherectomy is performed to achieve <30% residual stenosis and no flow-limiting dissection before DCB deployment [6,28]. This ensures the drug can contact the entire lesion and that no significant recoil remains after balloon deflation. If an extensive dissection (Type D or greater) or recoil occurs post-DCB, bailout stenting is performed to seal the artery. Thus, DCB in de novo lesions is often a DCB-first, stent-if-needed strategy. In trials, bailout stent rates vary (e.g., 7% in BASKET-SMALL2 [1] and ~20% in SELUTION DeNovo) [3]. Importantly, when bailouts are needed, the overall outcomes remain favorable, likely because DCB has already delivered the drug to the vessel, mitigating restenosis even in the stented segment. Another consideration is DAPT duration; after DCB-only treatment of a de novo lesion, guidelines generally recommend a shorter DAPT course (1 month) since there is no metal or polymer to protect [28]. This is a major advantage for patients at high bleeding risk (HBR). The DEBUT trial (2019) specifically studied HBR patients with de novo CAD, comparing paclitaxel DCB vs. bare-metal stents (BMS) to allow short DAPT. DCB was superior; 9-month target-lesion failure was 7% with DCB vs. 15% with BMS, and importantly, major bleeding was reduced by over 50% with the DCB strategy [29]. Although DES was not tested in DEBUT (DES would mandate longer DAPT, which HBR patients could not tolerate), the implication is that DCB can achieve revascularization with less bleeding risk. Current European guidelines accordingly endorse DCB (over BMS) in HBR patients who can only take short DAPT [7,28].

In summary, for stable de novo lesions, DCB-P has proven efficacy in small vessels and certain subsets, showing comparable TLR and MACE rates to DES [23,24]. DCB-S is on the cusp of wider adoption pending further trial results. The benefits of a DCB-only approach—i.e., no implant, potentially shorter DAPT and avoidance of late stent complications—must be weighed against the requirement for excellent acute results and the discipline to abandon DCB in favor of a stent if suboptimal expansion or dissections occur. With careful patient and lesion selection (e.g., lesions that angioplasty can fully expand without major dissection), a DCB strategy can be highly effective in CCS patients. Indeed, some high-volume centers in Europe have already adopted a DCB-first approach for suitable small vessel lesions, reserving DES for larger or more complex cases.

## 4. Drug-Coated Balloons in Acute Coronary Syndromes (ACSs)

Treating an unstable plaque in the setting of ACS (particularly STEMI) with only a balloon and no stent might seem counterintuitive, since stents tack up dissections and provide scaffolding to prevent acute closure. DESs are the guideline-recommended default in ACS because they reduce recurrent ischemia without increasing MI or mortality compared to balloon angioplasty [17]. However, primary stenting in ACS has downsides; stents implanted amidst thrombus can lead to malapposition, and DESs in MI have shown delayed endothelialization relative to stable lesions. These factors contribute to higher rates of stent thrombosis in ACS settings. Additionally, very late complications (stent fracture, neoatherosclerosis) remain a concern years after MI. Thus, a “leave-nothing-behind” approach in ACS—restoring flow with angioplasty and drug delivery but without permanent metal—is conceptually attractive [4]. The challenge is ensuring acute vessel stability.

Regarding evidence from clinical trials, early exploration of DCB in ACS was cautious. The multicenter DEB-AMI trial [30] evaluated adding a paclitaxel DCB after BMS in STEMI. It showed no improvement over BMS alone in late loss, and DES was still superior for restenosis reduction. However, the trial underscored that DCB could be used without safety issues in the acute setting. A landmark study in ACS was the REVELATION trial (Vos et al., 2019), which specifically examined a DCB-only strategy in STEMI [4]. In this single-center RCT (120 patients), following successful thrombus aspiration and pre-dilation, patients with <50% residual stenosis were randomized to paclitaxel DCB vs. DES [4]. The primary endpoint—fractional flow reserve (FFR) of the infarct artery at 9 months—was virtually identical: mean FFR 0.92 in DCB vs. 0.91 in DES (*p* non-inf < 0.001). Only one acute recoil requiring stenting occurred in the DCB arm. By 9 months, TLR rates were low and equal (one patient in each arm), and there were no differences in reinfarction or death. This demonstrated the feasibility of a DCB-only approach in STEMI when the lesion is well prepared. At 5-year follow-up, MACE remained low (5.4% DCB vs. 7.5% DES) and numerically lower with DCB [31], although not statistically different due to a small sample. Similarly, the PEPCAD NSTEMI trial (Scheller et al., 2020) randomized 210 patients with non-ST elevation MI to either stent (BMS/DES per operator) or paclitaxel DCB. At 1 year, MACE rates were comparable (~9% in both groups), confirming that DCB angioplasty is non-inferior to stenting in certain MI patients. It is worth noting that in PEPCAD NSTEMI, about 45% of patients in the DCB arm still received a bailout stent, reflecting the real-world challenge in ACS of achieving an optimal result without stenting [32].

Several small studies and registries have further supported DCB use in ACS. Gobić et al. (2017) conducted a feasibility study in primary PCI for STEMI: 30 patients were treated with paclitaxel DCB (with BMS bailout if needed) vs. 30 with DES [33]. Despite the high-risk setting, 6-month outcomes showed no significant differences in restenosis or MACE between DCB and DES groups.

**Figure 3:**
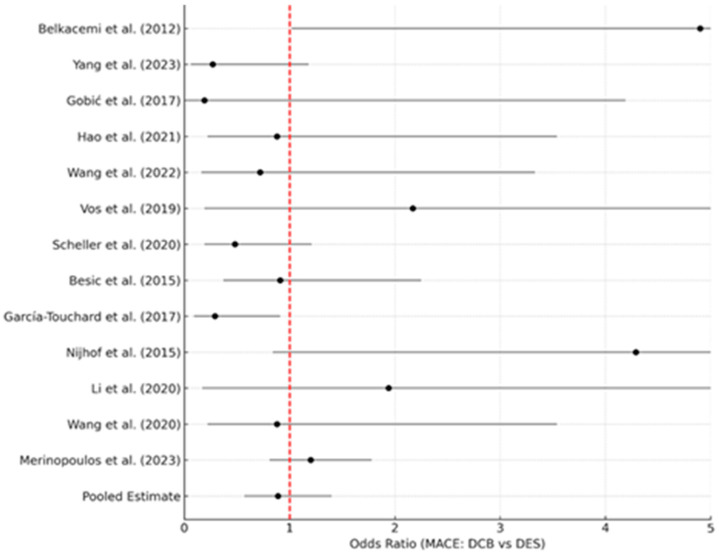
Representative forest plot adapted from the meta-analysis by Abdelaziz et al. (2023) [17], comparing drug-coated balloons (DCBs) and drug-eluting stents (DESs) in acute myocardial infarction.

Importantly, multiple meta-analyses now suggest that a DCB strategy in ACS is not inferior to DES in hard outcomes. Abdelaziz et al. (2023) pooled 13 studies (including REVELATION, PEPCAD, and others) comprising 2644 AMI patients [17]. They found no difference between DCB vs. DES in MACE (OR 0.89, 95%CI 0.57–1.40), all-cause mortality (OR 0.88, *p* = 0.73), reinfarction (OR 0.88, *p* = 0.79), or TLR (OR 0.90, *p* = 0.80). Interestingly, when MACE was defined more narrowly (cardiac death, MI, TLR), DCB was associated with significantly lower risk (OR ~0.50, *p* = 0.02). The authors attributed this to possibly fewer recurrent MIs in DCB-treated arteries (since there is no stent to thrombose or cause neoatherosclerosis). Figure 3 below shows a representative forest plot from their meta-analysis, demonstrating equivalent clinical outcomes between DCB and DES in AMI.

Another meta-analysis by Megaly et al. (2022) focusing on short-term outcomes in AMI also found DCB to be comparable to DES in death and TLR [34]. Notably, none of these analyses showed any signal of excess safety events with DCB; stent thrombosis was obviously eliminated, and acute vessel closures were very low with proper bail-out stenting protocols. That said, these studies were predominantly in lesions that were pre-selected or had met criteria (like residual stenosis <50% after ballooning) for safe deferral of stenting. DCB is clearly not suitable for every ACS lesion. Large thrombus burden, extensive dissection after dilation, or residual stenosis >30–50% are scenarios where a stent is still indicated in ACS [6]. The 2020 International DCB Consensus Group explicitly advises against DCB-only treatment in presence of “obvious angiographic thrombus” in ACS. They also emphasize that if flow-limiting dissection or recoil occurs, one should not hesitate to implant a stent. Essentially, lesion preparation and judgment are paramount in ACS: the operator must be ready to stent if an adequate result cannot be secured with the balloon alone.

### 4.1. ACS Clinical Outcomes and DAPT

In patients treated with DCB-only during ACS, a shortened DAPT strategy can be considered, though data are not as robust as in stable CAD. Many ACS-DCB studies still maintained standard DAPT for 6–12 months, since the patients had an acute MI (which itself is an indication for at least 12 months of antiplatelet therapy absent bleeding issues). However, if the patient is truly at high risk of bleeding, the ability to stop DAPT earlier after DCB (as no stent is present) is a significant advantage. For instance, in the aforementioned Merinopoulos 2023 series, some STEMI patients on DCB were able to de-escalate to single antiplatelet therapy after just 1 month without ischemic penalty [35]. This flexibility can be life-saving in ACS patients who develop bleeding or require urgent surgeries post-MI.

Major cardiology guidelines published since 2021 continue to favor drug-eluting stents (DESs) as the default strategy in acute coronary syndromes (ACSs), with drug-coated balloons (DCBs) remaining an investigational or niche option. For instance, the 2023 ESC Guidelines for ACS (covering both STEMI and NSTE-ACS) acknowledge emerging DCB data but stop short of recommending DCBs routinely. The ESC panel highlighted small trials (e.g., REVELATION and PEPCAD NSTEMI) where DCB-only PCI showed non-inferior short-term outcomes to DES in ACS; however, due to the limited sample sizes and follow-up, they concluded that “the use of DCB in NSTE-ACS requires further investigation” before it can inform formal recommendations [36]. In practice, this means DES implantation remains the standard of care in ACS per current guidelines, and DCB is not yet endorsed as a first-line therapy in myocardial infarction outside of specific research settings. Likewise, no major updates from ACC/AHA since the 2021 revascularization guideline have altered the stance; DES is still recommended by default for ACS, and DCB is generally reserved for special scenarios (such as in-stent restenosis or cases where stenting is not feasible) [37].

### 4.2. Recent Expert Consensus and Position Statements

While official guidelines are conservative, recent expert consensus documents have started carving out a role for DCB in carefully selected ACS cases. Notably, an Asia–Pacificic Consensus Group (2025) report provided practical guidance for a “DCB-based PCI” strategy, emphasizing optimal lesion preparation and using DCB as the default device with stents deployed only if necessary [38]. This consensus highlights that when certain criteria are met (well-prepared lesions with no flow-limiting dissection), DCB-only PCI can achieve outcomes comparable to DES, while conferring the known benefits of avoiding a permanent implant—no stent thrombosis risk, no long-term foreign body, and potentially fewer late adverse events. Similarly, other position papers have pointed out scenarios like high bleeding-risk patients in ACS, where a DCB-only approach may be advantageous by allowing much shorter DAPT duration. For example, a 2025 consensus statement from the Thai Cardiovascular Intervention Association explicitly states that “DCB angioplasty is an alternative option in HBR patients, [allowing] for shorter DAPT duration” [39]. These expert opinions echo the emerging evidence that in select patients (e.g., those who cannot tolerate prolonged dual antiplatelet therapy or have small-vessel disease), a stentless strategy can be considered.

In summary, the latest guidelines continue to recommend DES as standard in ACS, with DCB therapy not yet mainstream due to limited evidence. However, contemporary consensus statements reflect a growing enthusiasm for DCB in niche ACS settings, reinforcing the benefits of “leaving nothing behind” such as eliminating stent-related complications and simplifying post-PCI therapy [39,40]. It is expected that as ongoing trials report results, future guideline updates may incorporate more guidance on DCB use in acute MI, especially for patients meeting strict criteria for safe stent avoidance. For now, DES remains the default in ACS per guidelines, with DCB reserved for carefully selected cases based on expert consensus rather than broad guideline endorsement.

## 5. In-Stent Restenosis: DCB-S vs. DCB-P vs. DES for ISR

In-stent restenosis remains a challenging scenario, particularly after DES failure. The presence of a metallic stent cage limits repeat intervention options: placing additional layers of DES can increase late thrombosis risk and create a “full metal jacket”, whereas simply performing balloon angioplasty without addressing biological hyperplasia yields poor results. Drug-coated balloons offer an elegant solution for ISR by delivering the antiproliferative drug to the restenotic segment without adding another stent layer. Paclitaxel DCBs were first clinically proven in ISR and have since become an established therapy. The rationale is as follows: numerous trials and pooled analyses show DCBs produce equivalent (and sometimes superior) outcomes to repeat stenting in ISR, with the benefit of no extra metal and shorter required DAPT.

### 5.1. Paclitaxel DCB vs. DES in ISR

The evidence base is robust. The ISAR-DESIRE 3 trial (2013) was a pivotal randomized study in patients with DES-ISR [41]. At 3-year follow-up, the drug-coated balloon (PEB) showed similar target lesion revascularization rates compared with the paclitaxel-eluting stent (PES) and lower rates compared with plain balloon angioplasty (BA). The incidence of death or myocardial infarction was lower with PEB compared to PES, although this difference did not reach statistical significance (*p* ≈ 0.08); however, all-cause mortality was significantly reduced with PEB. Clinical outcomes were similar between PEB and BA. Notably, DCB performed as well as a paclitaxel-eluting stent in suppressing restenosis. Long-term follow-up revealed comparable clinical outcomes between the DCB and DES groups; however, DCB treatment was associated with fewer late complications. Notably, no stent thromboses occurred in the DCB arm (as no new stents were implanted), whereas several late stent thromboses were reported following repeat DES implantation. Another series of Spanish trials (RIBS IV and V, led by Alfonso et al.) also affirmed DCB efficacy in ISR. In RIBS IV (DES-ISR, n ≈ 309), EES achieved larger angiographic lumen (MLD 2.03 vs. 1.80 mm, *p* < 0.01) and fewer repeat revascularizations than DCB; overall outcomes favored EES (TLR 7.1% vs. 15.6%, *p* = 0.015) [42]. In RIBS V, DEB and EES had similar 1-year MACE (6% vs. 8%; HR 0.76, *p* = 0.6). EES produced better angiographic dimensions (MLD 2.36 vs. 2.01 mm, *p* < 0.001), though late lumen loss and binary restenosis were low and not significantly different [43]. The takeaway was that paclitaxel DCB offers similar efficacy to placing a second DES, without burdening the artery with more metal. A 2015 network meta-analysis by Siontis et al., integrating 27 ISR trials (5923 patients), ranked paclitaxel DCB and newer-generation DES as essentially equivalent for TLR reduction [44].

The largest evidence comes from the DAEDALUS meta-analysis (European Heart Journal 2020), which pooled individual patient data from 10 RCTs (n > 2000 ISR patients) [45]. At 3 years, DCB was associated with significantly higher TLR (TLR ≈16% vs. 12%; HR 1.32, *p* = 0.035), with a corresponding non-significant trend toward fewer MI or stent thrombosis (composite death/MI/TLT, HR 0.80, *p* = 0.15). Overall, repeat DES was “moderately more effective” in reducing revascularization. Additionally, the FDA-approved AGENT trial (Yeh et al., JAMA 2024) reinforced DCB benefits in ISR: in 600 patients with DES-ISR, the paclitaxel DCB (with new delivery catheter) was superior to POBA with respect to the composite end point of target lesion failure [46], leading to US approval of a coronary DCB.

### 5.2. Sirolimus DCB in ISR

Historically, virtually all ISR studies used paclitaxel DCBs. Sirolimus DCBs are now being tested. The first RCT in ISR with an SCB, called SELUTION4ISR (Cutlip et al., 2025), compared a sirolimus DCB vs. “standard of care” (which included mostly DES) in patients with ISR [5]. Presented as a TCT 2025 late-breaker, at 1 year, the SCB was non-inferior to repeat stenting; TLF was ~14% in both arms, with no safety concerns. As these data are currently available only from conference presentations, conclusions regarding clinical equivalence should await full peer-reviewed publication.

This suggests that sirolimus DCB can effectively treat ISR, although longer follow-up is awaited. Another question is whether sirolimus offers any advantage in ISR over paclitaxel. Theoretical benefits include less cytotoxic injury to an already diseased vessel and possibly better endothelial recovery inside the stent. However, limited data (e.g., small observational studies) indicate no significant differences in angiographic or clinical ISR outcomes between SCBs and PCBs. Paclitaxel’s edge in tissue penetration might be less of an issue in ISR, since the drug can more easily diffuse through the stent struts and thin neointima. Thus, while SCBs provide an alternative (especially useful if paclitaxel DCB is contraindicated or unavailable), paclitaxel DCB remains the workhorse for ISR at present.

### 5.3. DES-in-DES vs. DCB

When a patient develops restenosis in a DES, a common strategy is to place another DES (often a different drug)—so-called “DES-in-DES” treatment. This can be effective but comes with cumulative metal layers. Studies comparing this to DCB are insightful. In ISAR-DESIRE3 and RIBS IV, paclitaxel DCB performed on par with second DES [41,43]. The DARE trial (Baan et al., 2018) specifically randomized any ISR (DES or BMS) to paclitaxel DCB vs. everolimus DES [47]. At 12 months, there was no significant difference in ISR recurrence and target vessel revascularization. DARE found non-inferior 6-month MLD (1.71 vs. 1.74 mm, *p* < 0.001 noninferiority) and similar 1-year TVR (8.8% DEB vs. 7.1% DES, *p* = 0.65. Notably, in DES-ISR subsets, outcomes were almost identical between DCB and DES. This trial reinforced that avoiding another stent does not compromise efficacy in ISR. The decision between DCB vs. DES for ISR can thus be individualized—DCB is generally preferred if the lesion can be fully covered by the balloon and there is no contraindication to paclitaxel, whereas placing a new DES might be chosen if there was an underlying mechanical issue (e.g., stent underexpansion that a new stent with high pressure could fix). In fact, imaging-guided studies show that many ISR cases involve stent underexpansion; in those, optimal lesion prep (e.g., high-pressure dilatation, scoring/cutting balloon) followed by DCB yields excellent results [44].

### 5.4. DCB for ISR—Practical Use

In both BMS-ISR and DES-ISR, DCB angioplasty technique mirrors that for de novo lesions; the lesion is aggressively prepared (often including debulking the neointimal tissue with cutting balloons or rotablator in very tough ISR) and ensuring the balloon can fully expand the stent to its nominal diameter. Imaging (IVUS/OCT) is helpful to identify any mechanical cause (underexpansion or fracture) that should be corrected (e.g., with high-pressure non-compliant balloon or scoring balloon) before DCB drug delivery. After DCB inflation, one must be vigilant for elastic recoil—it is generally less problematic within stents, but edge dissections can occur. If significant, spot-stenting (with a short DES) can be carried out, though this somewhat defeats the “no-metal” intent. In the largest DCB ISR studies, bailout stenting rates were low (<7%) [24]. DAPT duration after DCB for ISR is usually 6 months (if it is DES-ISR, the initial DES’s endothelialization is presumably complete; if it is BMS-ISR, only a bare stent remains, which is low-risk). Some physicians even treat DCB in ISR like plain balloon in terms of DAPT—e.g., a month of DAPT is considered sufficient by the consensus if no new stent is placed [6].

In summary, paclitaxel DCB is a first-line therapy for ISR, supported by multiple RCTs and meta-analyses [41,43,45]. It achieves similar restenosis rates to placing another DES, without adding layers of metal that could complicate future interventions or surgery. Sirolimus DCBs are emerging in this space and appear effective, though long-term comparative data are pending. A practical approach is as follows: for ISR lesions that are diffusely proliferative (true restenosis) without significant stent malapposition or mechanical issues, DCB is ideal; for ISR caused by focal mechanical problems (stent collapsed, etc.), fixing with another stent or scoring balloon plus DCB might be needed. Table 2 highlights key studies in ISR management.

The table underscores that DCB has consistently matched DES in ISR trials, achieving comparable LLL and repeat revascularization rates, whether the ISR originated in a BMS or DES. DES-in-DES is not clearly superior and may be inferior in some respects (repeat revascularization risk, etc.). Paclitaxel DCBs have the longest track record in ISR, but sirolimus DCBs are likely to be included in future updates of guidelines once more data (like SELUTION4ISR) are published [5]. Given this evidence, current practice patterns have shifted: many operators now prefer DCB for diffuse ISR, reserving repeat DES for very focal ISR or cases needing additional scaffold.

## 6. Guideline Indications and Emerging Use-Cases for DCBs

Recent practice guidelines and consensus papers have begun to acknowledge drug-coated balloons (DCBs) as an alternative in selected patients, but DES remains the default in ACS. For example, the 2023–2024 European Society of Cardiology (ESC) guidelines emphasize stenting for ACS but explicitly include DCB as an option in certain settings. In particular, the 2024 ESC Chronic Coronary Syndrome guideline gives a Class IA recommendation to treat stent restenosis with either a new-generation DES or a DCB [7] (notably, the analysis of RIBS trials cited in the ESC guidelines showed DCB and even bioresorbable scaffolds were inferior to DES for ISR [48]). The ESC guidelines do not mandate DCB in primary ACS, but they do recognize DCB (with optimal lesion prep) as reasonable in scenarios like restenosis or small-vessel disease. By contrast, US ACC/AHA guidelines have not been updated since the 2015–2020 era for ACS and still implicitly favor DES (no new recommendation to use DCB in ACS has appeared) [37]. Importantly, guideline recommendations may endorse more than one effective therapeutic option (Class I), even when comparative trials or meta-analyses suggest modest differences between strategies. In this context, the Class I recommendation for DCB in ISR reflects consistent efficacy and safety across multiple studies, while observed differences versus DES relate to specific endpoints and do not preclude guideline endorsement.

- ESC 2023–2024: The new ESC ACS guidelines (2023) and the 2024 chronic CAD guidelines reaffirm stenting for acute MI but do mention DCB use in niche cases. For instance, the ESC notes that treating ISR may use either DES or DCB (Class I), and it discusses DCB for small-vessel or bifurcation lesions in chronic CAD. Overall, ESC still highlights DES as preferred for STEMI/NSTEMI but acknowledges DCBs “leave nothing behind” PCI in select lesions [7,36].
- ACC/AHA: The latest American guidelines on PCI/ACS (no major update since 2020) continue to default to DES in ACS. There is currently no new ACC/AHA recommendation endorsing routine DCB in STEMI/NSTEMI; ACS patients who meet strict criteria (e.g., very small vessel, high bleeding risk) remain a niche group. In short, U.S. guidelines have not changed—DES are still the first-line treatment in ACS [37] (by implication, the benefits of DCB cited—no scaffold, lower late events—are still considered experimental in ACS.)
- Asia–Pacificic Consensus (2025): The Second Asia–Pacificic DCB Consensus (JACC: Asia 2025) explicitly promotes a “DCB-first” strategy in prepared lesions. It advocates a provisional approach (“all coronary lesions are treated using the provisional approach, where active lesion preparation is followed by DCB or DES treatment”), effectively making DCB the default device before starting intervention [38]. In this view, the operator begins with optimal vessel preparation and uses DCB if results are acceptable, reserving DES only for bailout. This consensus document (led by the Asia–Pacificic consensus group) provides practical guidelines on DCB-based PCI across various lesion types.
- Key takeaway: In 2024–2025, DES remains the cornerstone for ACS in guidelines, but DCB is increasingly recognized in “leave-nothing-behind” strategies. Updated ESC/ACC guidelines have not overruled DES-first for ACS, yet they do permit DCB in special situations (especially ISR) [7,37]. Newly issued consensus statements (Asia, Japan, etc.) explicitly endorse DCB as an alternative in select ACS/CAD cases [38]. Thus, the “niche role” of DCB in acute MI—avoiding a permanent implant in very specific scenarios—is now supported by multiple position papers, even if formal guideline classes remain conservative.

Professional consensus documents provide more practical guidance on DCB use. The International DCB Consensus (third report) gave detailed recommendations, detailed as follows [6].

- ISR: DCB is suitable for both BMS and DES restenosis. Good candidates are those with first-time ISR, multiple stent layers (to avoid a third), ISR involving a side-branch (so it is not jailed with another stent), or HBR patients where avoiding long DAPT is crucial. If the ISR has a clear mechanical cause (like stent underexpansion), they advise correcting that before DCB.
- Small vessels: DCB has relevant evidence in vessels ≤2.75 mm or <3.0 mm in de novo lesions, with at least two RCTs showing non-inferiority to DES(BASKET-SMALL2 and PICCOLETO II) and even a trend toward less late loss [23,24]
- Large vessels: In vessels >3 mm, the consensus notes that randomized data are limited. Thus, in large vessels, DCB can be used (especially if the lesion is short or discrete), but robust evidence is still emerging.
- Bifurcation lesions: DCB can be utilized to avoid complex two-stent techniques. The consensus recommends the provisional stent strategy for the main branch and suggests that for the side branch, DCB angioplasty (instead of plain balloon) “may be preferable” to reduce ostial restenosis. Clinical data (from registries and small trials) have shown that in bifurcation PCI, using a DCB on the side branch after main vessel stenting yields better side branch patency than balloon alone and is far simpler than putting two stents. Ongoing trials like DEBUT-BIF are examining this formally [49].
- Diabetes mellitus: Guidelines note that diabetic patients have higher restenosis rates. While DESs are standard in diabetics, the consensus opines that DCBs “may be a favorable alternative” in certain diabetic subsets, but more data are needed. Notably, a subanalysis of BASKET-SMALL2 found no interaction of diabetes status—DCB was as effective as DES in diabetics for small vessels, though absolute event rates were higher than in non-diabetics [23].
- High bleeding risk: Both consensus and guidelines highlight HBR as a prime indication for DCB. If a patient cannot tolerate >1 month of DAPT, a DCB-only strategy is very attractive, since only 4 weeks of DAPT is recommended after DCB in de novo lesions. The DEBUT trial’s positive results in HBR support this [29]. Thus, for an HBR patient with a moderate lesion that can be ballooned-open nicely, one might use a DCB and give 1 month of DAPT, as opposed to using a DES with 3–6+ months DAPT and attendant bleeding risk.

## 7. Emerging Indications for Sirolimus DCBs

With the advent of effective sirolimus-coated balloons, one can envision expanding DCB usage to patients and lesions where paclitaxel DCBs had limitations. Some examples are detailed below.

- Patients reluctant to accept any theoretical paclitaxel risks: While no mortality signal was ever seen in coronary trials (in contrast to a controversial signal in peripheral arterial disease), some patients/providers are more comfortable with limus drugs (given their long safety record in stents). SCBs offer an alternative in such cases. The consensus explicitly noted that no paclitaxel-related mortality concerns have emerged in coronaries, and 2-year data show no increase in death with DCB vs. DES, but it is reassuring that SCBs exist as an option [6].
- Very long lesions/diffuse disease: Tacking multiple long DES segments can lead to an excess of metal, overlapping polymers, etc. A “full metal jacket” can impair vessel compliance and complicate future grafting. DCBs could treat long diffuse disease by serially delivering the drug along the artery. This strategy is still investigational, but SCBs with sustained release might especially help in long lesions by providing a longer antiproliferative effect akin to overlapping DES.
- SCB for ISR in limus–DES failures: Conceptually, treating restenosis of a limus-eluting stent with another limus source (SCB) could be advantageous to maintain the same drug (avoiding potential cross-resistance issues). Paclitaxel DCBs are effective regardless of the stent type (limus or paclitaxel DES), but SCBs provide another tool. The ongoing TRANSFORM-1 and -2 trials are examining SCB in ISR and de novo lesions, respectively, to solidify its role [27].
- Combining with bioresorbable scaffolds: A novel concept is using a bioresorbable scaffold (BRS) in one part of a lesion and a DCB in the adjacent segment, to minimize permanent material. BRS can address a focal high-risk segment (providing early support), and DCB treats the rest diffusely. As next-generation BRSs improve, SCBs may complement them; both leave behind no permanent metal in the long term.
- Adjunct to atherectomy: After orbital, rotational, or laser atherectomy in a calcified lesion, one might consider DCB if the vessel is well prepared and perhaps avoid a stent, especially in moderate calcification. However, heavily calcified lesions often need stents to maintain expansion, so this is a very niche application currently.

## 8. Limitations of Current Data and Ongoing Research

An additional limitation of several DCB trials, particularly in ACS and de novo lesion settings, is the potential for selection bias. In many studies, only lesions achieving an optimal angiographic result after lesion preparation were eligible for randomization to a DCB strategy. This design may limit the generalizability of trial results to real-world practice, where lesion preparation may be suboptimal or less predictable.

Despite promising results, many DCB trials have been relatively small or with angiographic endpoints. The SELUTION DeNovo trial is a game-changer in terms of scale, but full peer-reviewed publication is awaited. Longer-term outcomes (beyond 1–3 years) for DCB vs. DES need to be delineated—it is possible that DCB’s “exit strategy” of leaving nothing behind results in fewer very-late events (e.g., no late stent thrombosis, and less late neoatherosclerosis) [3]. Indeed, PICCOLETO II’s late follow-up hinting at lower 3-year MACE with DCB is intriguing, as was a mortality advantage for DCB at 3 years in one meta-analysis. However, these are hypothesis-generating. Conversely, one must watch for any late catch-up in DCB-treated lesions, but so far, trials like BASKET-SMALL2 at 3 years show stability [23]. Another limitation is that most DCB studies used surrogate endpoints (LLL, % stenosis); while these correlate with outcomes, definitive trials powered for clinical endpoints are fewer. The ongoing UK DCB trial and others may help answer this by randomizing broader populations to DCB vs. DES with clinical primary endpoints.

From a methodology perspective, DCB trials face the challenge of unblinded angiographic follow-up; knowledge of treatment could bias revascularization decisions. Some trials mitigated this by using FFR or imaging endpoints (e.g., REVELATION used FFR [31]. Future studies employing sham control (like a “placebo” balloon vs. DCB) would be ideal but hard to execute. The field is also advancing with new endpoints; for instance, the concept of “vessel healing” measured by OCT could one day become a way to compare DCB vs. DES beyond just lumen diameter.

Recent innovations: DCB technology is evolving beyond just the drug. Research is underway on dual-drug balloons (e.g., combining an anti-proliferative with an anti-inflammatory or endothelial progenitor-capturing agent) to further improve outcomes in complex lesions [50]. There is interest in using pharmacologic agents like NO-donors or fibrinolytics on balloons in ACS to address thrombus and endothelial function. Another innovation is liquid sirolimus balloons—a recent preclinical study (Todd et al. 2023) tested a balloon that releases sirolimus in liquid formulation for deeper vessel penetration [51]. In the realm of procedural planning, AI and advanced imaging are being applied to optimize lesion prep strategies for DCB (e.g., algorithms to predict which lesions can be safely treated without a stent). These adjuncts could increase DCB success rates by better case selection and real-time guidance.

Throughout this review, evidence derived from fully published randomized controlled trials is clearly distinguished from late-breaking or conference-presented data, which are discussed as emerging and hypothesis-generating.

## 9. Conclusions

In conclusion, sirolimus-coated DCBs are poised to expand the DCB paradigm, building on the strong foundation laid by paclitaxel DCBs. Current evidence and guidelines already support DCB (especially DCB-P) in ISR and small vessels, and use in de novo CAD is growing as large trials demonstrate non-inferiority to DES [5]. In ACS, DCB-only PCI is an intriguing strategy that challenges the conventional need for a stent in every infarct. Studies show it can work in carefully selected cases with diligent technique [17]. Sirolimus DCBs, with improved delivery kinetics, have shown for the first time that a limus-drug balloon can effectively compete with DES in broad populations [3]. They offer the possibility of a metal-free intervention with a drug most interventionalists are already comfortable with (as used on DES). As more data emerge, we may see guideline updates granting DCB-S a role alongside DCB-P. Ultimately, the interventional community is moving toward an era of precision PCI, selecting the right tool (DES, DCB-P, or DCB-S) for each lesion and patient. In this context, drug-coated balloons (of either drug) are an increasingly important part of the armamentarium, enabling effective revascularization while leaving nothing behind except the drug’s effect. The continued refinement of DCB technology and technique holds the promise of improving long-term outcomes and freedom from reinterventions for our patients with coronary artery disease.

## Figures and Tables

**Table 1:** Pharmacological profile of paclitaxel and sirolimus drug-coated balloons [15,16].

Property	Paclitaxel (DCB-P)	Sirolimus (DCB-S)
Mechanism of Action	Microtubule stabilizer; induces G_2_/M arrest (cytotoxic).	mTOR inhibitor; induces G_1_ arrest (cytostatic).
Lipophilicity (log *p*)	High (~3.9)—facilitates rapid tissue uptake and long retention.	Moderate (~2.5)—lower innate uptake; needs delivery enhancements.
Tissue Retention	Prolonged (weeks). The drug persists in the vessel wall after a single use.	Shorter without special excipients. Advanced coatings prolong release to ~60–90 days.
Endothelial Healing	Slower re-endothelialization; moderate chronic inflammation.	Faster endothelial recovery; lower inflammatory response in studies.
Clinical Usage (2025)	Decade-long track record; gold-standard DCB agent.	Emerging in trials, promising results with new delivery technologies.

**Table 2:** Key studies of DCB vs. DES in coronary in-stent restenosis. Abbreviations: POBA = plain old balloon angioplasty; %DS = percent diameter stenosis; ns = not significant.

Trial	Population (ISR Type, Prior Stents)	Intervention vs. Comparator	Follow-Up	Angiographic Findings	Clinical Outcomes (1 yr Unless Noted)
ISAR-DESIRE 3 (2013) [41]	DES–ISR (≥50% restenosis after “limus”-eluting stents); N = 402	Paclitaxel DCB (PEB) vs. Paclitaxel DES (PES) vs. POBA	6–8 mo angiography; 1 yr clinical	6–8 mo % diameter stenosis: PEB 38.0% vs. PES 37.4% (Δ0.6%, one-sided *p*_non-inf = 0.007); both PEB and PES vs. BA (54.1%, *p* < 0.0001).	Death, MI, and target-lesion thrombosis were similar across arms; 1 yr TLR (“repeat revascularization”) was similar for PEB vs. PES but both lower than POBA.
RIBS IV (2015) [43]	DES–ISR; N = 309 (DEB 154 vs. EES 155)	Paclitaxel DCB (DEB) vs. Everolimus DES (EES)	6–9 mo angiography; 1 yr	6–9 mo minimal lumen diameter: EES 2.0 ± 0.7 mm vs. DEB 1.80 ± 0.6 mm (*p* < 0.01); %DS 23 ± 22% vs. 30 ± 22% (*p* < 0.01); binary restenosis 11% vs. 19% (*p* = 0.06).	1 yr composite (cardiac death/MI/TVR): 10% (EES) vs. 18% (DEB), HR ≈ 0.58 (95% CI 0.35–0.98; *p* = 0.04); mainly driven by TVR (8% vs. 16%, *p* = 0.035).
RIBS V (2014) [42]	BMS–ISR; N = 189 (DEB 95 vs. EES 94)	Paclitaxel DCB (DEB) vs. Everolimus DES (EES)	9 mo angiography; 1 yr	9 mo MLD: EES 2.4 ± 0.6 mm vs. DEB 2.0 ± 0.6 mm (*p* < 0.001); %DS 13 ± 17% vs. 25 ± 20% (*p* < 0.001). Late loss low in both (0.04 vs. 0.14 mm, *p* = 0.14); restenosis ~4.7% vs. 9.5% (*p* = 0.22).	1 yr MACE (cardiac death/MI/TVR): 6% (EES) vs. 8% (DEB), HR≈0.76 (*p* = 0.6); TVR: 2% vs. 6% (HR ≈ 0.32, *p* = 0.17). Both groups had very low restenosis and high patency.
DARE (2018) [47]	Any ISR (BMS or DES; 56% DES); N = 278 (PEB 141 vs. EES 137)	Paclitaxel DCB (SeQuent Please) vs. Everolimus DES (Xience)	6 mo angiography; 12 mo	6 mo in-segment MLD: DCB 1.7 ± 0.51 mm vs. DES 1.7 ± 0.61 mm; noninferior (*p* < 0.0001 for noninferiority). Post-procedure, DES had slightly larger MLD and lower %DS than DCB (*p* = 0.018 and *p* = 0.03).	1 yr TVR: 8.8% (DCB) vs. 7.1% (DES) (*p* = 0.65); no significant differences in MACE (death/MI/TVR) between groups.
DAEDALUS (2020) [45]	Meta-analysis of 10 RCTs in ISR (BMS or DES); total N = 1976 (PCB n = 1033 vs. DES n = 943)	Paclitaxel-coated balloon angioplasty vs. repeat DES	Median 3 yr	–	3 yr TLR: higher with DCB (PCB) vs. DES (HR 1.32; 95% CI 1.02–1.70; *p* = 0.035). The DCB–DES difference was greater for DES-ISR (treatment-by-ISR-type interaction *p* = 0.029). Safety composite (all-cause death/MI/stent thrombosis) was similar (HR 0.80; *p* = 0.152), as were mortality, MI, and thrombosis.
AGENT IDE (2024) [46]	Any coronary ISR (lesion ≤ 26 mm, RVD 2.0–4.0 mm); N = 600 (2:1 randomization)	Paclitaxel DCB (AGENT balloon) vs. uncoated balloon (POBA)	1 yr	–	1 yr TLF (ischemia-driven TLR, TV-MI, or cardiac death): 17.9% (DCB) vs. 28.6% (POBA), HR 0.59 (95% CI 0.42–0.84; *p* = 0.003). Ischemic TLR: 13.0% vs. 24.7%, HR 0.50 (*p* = 0.001). TV-MI: 5.8% vs. 11.1% (HR 0.51, *p* = 0.02). Stent thrombosis: 0.4% vs. 3.7% (HR 0.07, *p* = 0.001). Death: 4.1% vs. 3.7% (*p* = 0.85).

## Data Availability

No new data were created or analyzed in this study.

## Abbreviations

The following abbreviations are used in this manuscript:

ACS	Acute Coronary Syndrome
BA	Balloon Angioplasty (Plain Old Balloon Angioplasty, POBA)
BMS	Bare-Metal Stent
CAD	Coronary Artery Disease
DAPT	Dual Antiplatelet Therapy
DCB	Drug-Coated Balloon
DES	Drug-Eluting Stent
EES	Everolimus-Eluting Stent
HR	Hazard Ratio
ISR	In-Stent Restenosis
LLL	Late Lumen Loss
MACE	Major Adverse Cardiac Events
MI	Myocardial Infarction
MLD	Minimal Lumen Diameter
OR	Odds Ratio
PEB	Paclitaxel-Eluting Balloon (another term for paclitaxel-coated DCB)
PES	Paclitaxel-Eluting Stent
POBA	Plain Old Balloon Angioplasty (also referred to as BA)
RCT	Randomized Controlled Trial
SCB	Sirolimus-Coated Balloon
TLF	Target Lesion Failure
TLR	Target Lesion Revascularization
TVF	Target Vessel Failure
TVR	Target Vessel Revascularization
